# Image Montaging for Creating a Virtual Pathology Slide: An Innovative and Economical Tool to Obtain a Whole Slide Image

**DOI:** 10.1155/2016/9084909

**Published:** 2016-09-22

**Authors:** Spoorthi Ravi Banavar, Prashanthi Chippagiri, Rohit Pandurangappa, Saileela Annavajjula, Premalatha Bidadi Rajashekaraiah

**Affiliations:** ^1^Oral Diagnostic and Surgical Science Division, International Medical University, No. 126, Jalan 19/155B, 57000 Bukit Jalil, Kuala Lumpur, Malaysia; ^2^Oral Pathology and Oral Medicine Division, Faculty of Dentistry, MAHSA University, Bandar Saujana Putra, 41200 Jenjarom, Selangor, Malaysia; ^3^Restorative Dentistry Division, International Medical University, No. 126, Jalan 19/155B, 57000 Bukit Jalil, Kuala Lumpur, Malaysia; ^4^MDS, Oral and Maxillofacial Pathology, 12-13-36, Lakshmi Nivas, Tarnaka, Hyderabad 500017, India; ^5^Oral Pathology, JSS Dental College and Hospital, Sri Shivarathreeshwara Nagara, Bannimantap, Mysore 570015, India

## Abstract

*Background*. Microscopes are omnipresent throughout the field of biological research. With microscopes one can see in detail what is going on at the cellular level in tissues. Though it is a ubiquitous tool, the limitation is that with high magnification there is a small field of view. It is often advantageous to see an entire sample at high magnification. Over the years technological advancements in optics have helped to provide solutions to this limitation of microscopes by creating the so-called dedicated “slide scanners” which can provide a “whole slide digital image.” These scanners can provide seamless, large-field-of-view, high resolution image of entire tissue section. The only disadvantage of such complete slide imaging system is its outrageous cost, thereby hindering their practical use by most laboratories, especially in developing and low resource countries.* Methods*. In a quest for their substitute, we tried commonly used image editing software Adobe Photoshop along with a basic image capturing device attached to a trinocular microscope to create a digital pathology slide.* Results*. The seamless image created using Adobe Photoshop maintained its diagnostic quality.* Conclusion*. With time and effort photomicrographs obtained from a basic camera-microscope set up can be combined and merged in Adobe Photoshop to create a whole slide digital image of practically usable quality at a negligible cost.

## 1. Background

Traditionally, education and training in pathology has been delivered using textbooks, glass slides, and conventional microscopy. Over the last two decades, the number of web-based pathology resources has expanded dramatically [1]. Whole slide imaging (WSI) technology permits glass slides to be scanned and viewed on a computer screen. This technology is referred to as virtual microscopy (VM). Technology for acquisition of virtual slides was developed in 1985; however, it was not until the late 1990s that the computers had enough processing speed to commercialize virtual microscopy and apply the technology to education. As technological advancements are happening at the speed of light, surveys now indicate that about 50% of pathology courses already have or expect to implement virtual microscopy [2] at least in the west.

High resolution pathology digital images provide huge information about the morphological and functional characteristics of biological systems and are transforming the field of pathology into a new era. Transition of digital pathology in clinical diagnosis has only begun recently. Digital pathology images, such WSI generated by scanning microscope slides, at diagnostic resolution enable VM of tissue specimens to support clinical diagnosis and biomedical research [3]. In reality a virtual pathology slide is a microscope emulator that presents via a computer [4].

The applications of WSI are tremendous, but, currently, the primary utilization of the technique is for transmission of digital images, second opinion consultation [5], quality assurance [6], teaching [7] and research [8], remote frozen section diagnosis [5], proficiency testing [6], multicenter research [5], and archiving [9]. The high resolution of digital images along with the refinement of technology could now allow for WSI to be used as an alternative to conventional microscopy (CM) [10]. The downside of such dedicated whole slide scanners is their sky high cost, which none of the pathology laboratories in developing countries can afford.

Currently, industry leading image editing software “Adobe Photoshop” is a household name. It has numerous features which can be employed in image processing and even basic analysis. In our quest to find an alternative to expensive slide scanners, we tried using the Adobe Photoshop's photo merge tool to create practically a collage of numerous photomicrographs of the given section to obtain a digital image of entire tissue section.

## 2. Materials and Methods

### 2.1. Construction of Virtual Pathology Slide (VPS)

**Figure 1:**
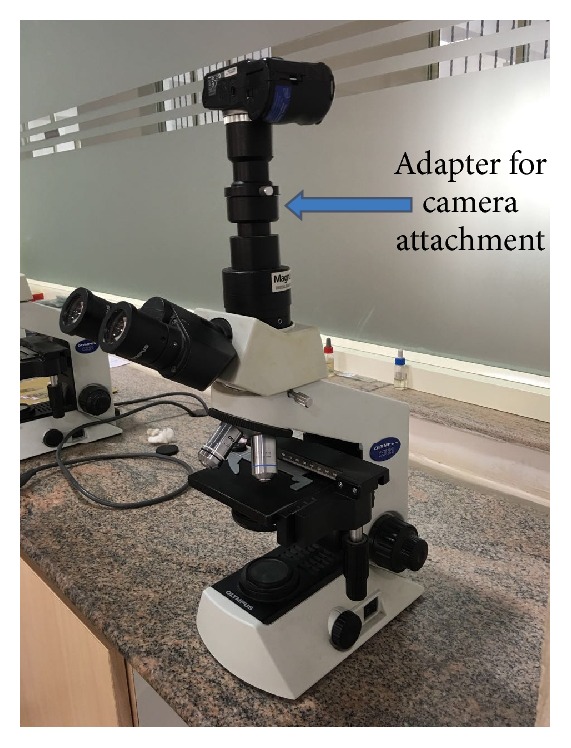
Basic setup required for capturing photomicrographs. Note the camera attachment adapter for attaching digital camera to the microscope.

A basic imaging workstation consisting of routinely used Olympus CX21 trinocular microscope (Olympus, Melville, NY, USA) with Olympus SP350 digital camera attachment was used to capture multiple images of randomly selected H&E stained routine running slides. The camera attachment to the microscope is through an Olympus provided custom made adapter with dedicated built-in optics, usually a 10x objective for most digital cameras (the lens is usually fixed inside such a position, such that focus is achieved, Figure 1). Camera is connected to a high specification computer with Adobe Photoshop CS3 software installed in it. 

*Certain Guidelines Were Followed to Capture and Transfer the Photomicrographs of Sections*

**Figure 2:**
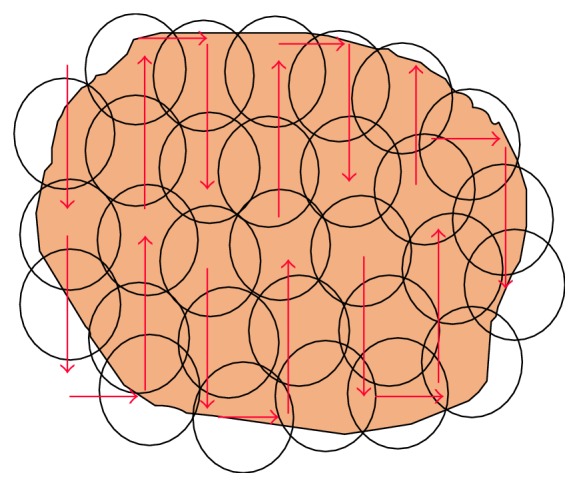
“Step ladder” pattern is followed during capturing process. Each circle represents one eyepiece view. Multiple overlapping circles covering the entire tissue section are captured.

1. Sections with artifacts, improper staining, multiple bit tissue sections, and shredded sections were not included. Instead, a nicely stained single bit tissue section was considered.
2. Using 10x objective, all areas of the entire section were photomicrographed in a step ladder fashion to make sure that no area is missed. To capture the photomicrograph, once the area to be captured is focused by looking on the computer monitor, if satisfied, then that area of the section can be captured. This has to be repeated for the entire section. The number of photomicrographs can vary from few 30–40 to hundreds depending on the size of the tissue section. Multiple “circles” of photomicrographs were finally obtained (note: multiple overlapping areas of photomicrographs are acceptable; in fact they are preferred, Figure 2). Photomicrographs of the tissue section can be captured under each objective separately if one wants WSI of each objective zoom level. (capturing multiple photomicrographs under 10x, 20x, and 40x) to get WSI of 10x, 20x, or 40x. The photomicrographs capturing process is conducted by an individual and software has no control over the capturing process in our technique because of our manual approach. If automation is achieved, software can be employed to do the same, thereby reducing the effort, but increases the cost of the equipment tremendously.
3. High specification desktop computer with a high speed processor coupled with a minimum of 2–4 GB RAM and loads of empty hard disk space is preferred to have a fast workflow.

The obtained images were then transferred to a computer with Adobe Photoshop CS3 Extended software installed. Photomicrographs were then opened in Adobe Photoshop CS3 Extended and Adobe Bridge for image optimization, merging, and creating a virtual pathology slide.

### 2.2. Actual Workflow in Adobe Photoshop in Creating VPS

#### 2.2.1. Premontage Image Optimization

One image will be used and the same settings will be applied to the rest of the images.

**Step 1 (handling JPEG file in Adobe Camera Raw (ACR)).**

**Figure 3:**
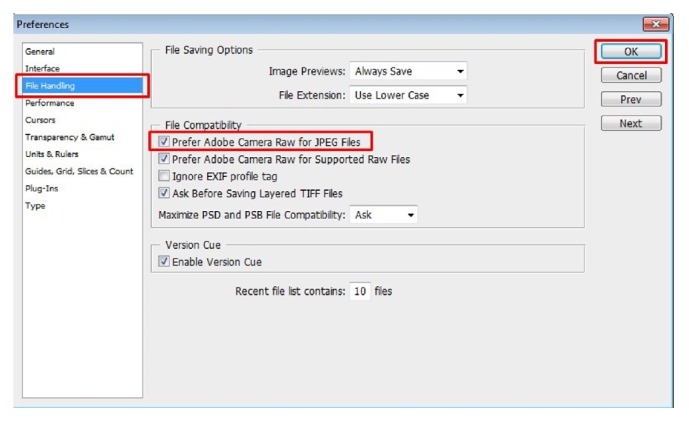
Change “settings” in photoshop (Edit > Preferences > File Handling) to open JPEG files in ACR. Note the red boxes in the figure. Follow them for an easy workflow.

To manage multiple images at once and thereby reducing much time, use Adobe's Bridge along with Adobe Photoshop. Adobe Bridge is an add-on application which comes with Adobe Photoshop. To use Adobe Bridge effectively and handle “JPEG” files as “Raw” file which makes JPEG file as a negative film of traditional camera and allows nondestructive image processing so one can do necessary modifications without affecting its originality, one has to change some “settings” in Adobe Photoshop. To do this, open Adobe Photoshop and click on “Edit” tab and select preferences under drop down menu to obtain a popup window. Select “File Handling” in that window and check “prefer Adobe Camera Raw for JPEG file” and select Ok (Figure 3). This makes Adobe Photoshop open even JPEG files in ACR.

**Figure 4:**
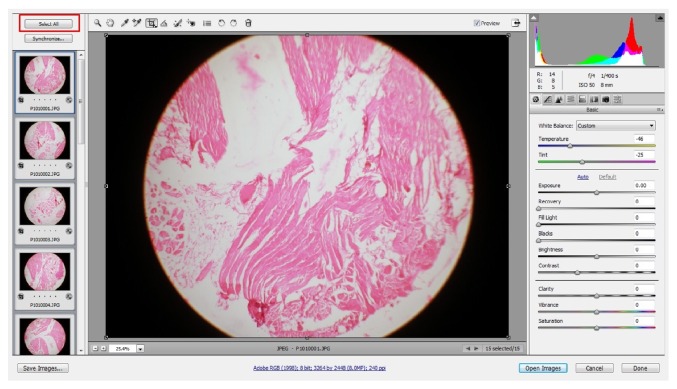
All photomicrographs opened in ACR window of Adobe Photoshop through Adobe Bridge. Note: select all to apply image optimizing settings to all images instantaneously. Note the red boxes in the figure. Follow them for an easy workflow.

Next, open Adobe Bridge and browse the folder where all the photomicrographs are located and select “all images” and right click on them and click on open in Adobe Photoshop. This command opens all the photomicrographs in ACR window through Adobe Photoshop (Figure 4).

**Step 2 (correcting the white balance).**

**Figure 5:**
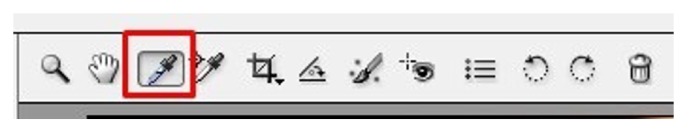
White Balance tool located at top left corner in ACR window. Note the red boxes in the figure. Follow them for an easy workflow.

Next, select all images in file viewer on the left in ACR window and then use “white balance” tool located on top left area of ACR window (Figure 5). Once the white balance tool is selected move into an area that should have no colorcast. In this case the slide background should be white, so with white balance tool selected click on any white appearing area to apply custom white balance. One can actually play with many other options available on the right side of ACR window to obtain desirable changes in image. Once white balance is corrected, click on “synchronize” to apply the same settings to all other photomicrographs instantaneously and hit on “done” to exit from ACR window.

**Step 3 (nondestructive cropping).**

**Figure 6:**
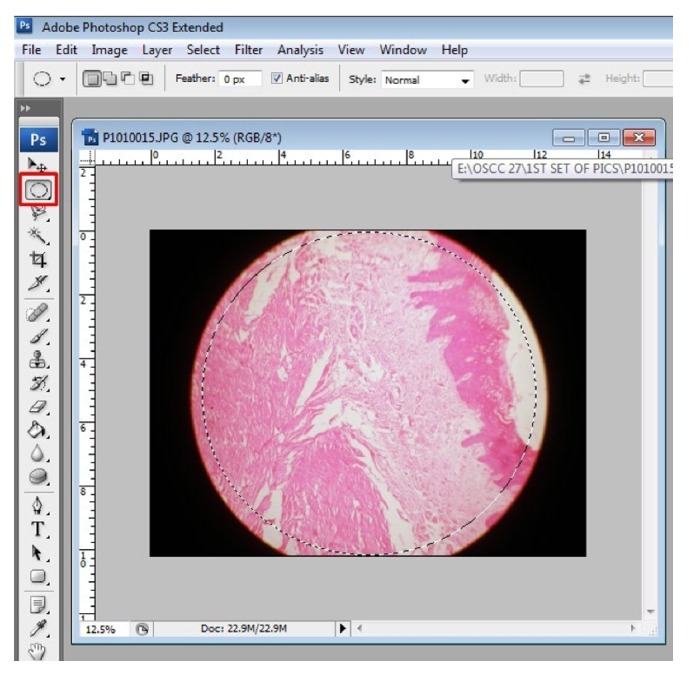
Select elliptical marquee tool and drag and draw a dotted line circle almost covering only the section and leaving out extraneous black portion. Note the red boxes in the figure. Follow them for an easy workflow.

**Figure 7:**
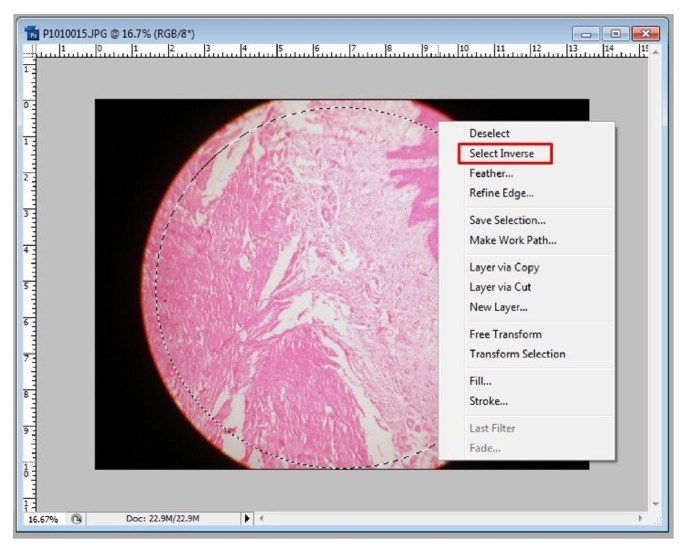
“Inverse selection” to delete extraneous portion of an image. Note the red boxes in the figure. Follow them for an easy workflow.

Before one can actually do this, reverse the “settings”; that is, change what was done initially to handle JPEG files in ACR. To simply do this, in Photoshop go to Edit > Preferences > File Handling > uncheck “Prefer Adobe Camera Raw for JPEG Files” and hit Ok. From now onwards each image has to be opened in Photoshop separately. Go to file > open > browse to the location of file and click on it to open in Photoshop window. To obtain only the required part of the image and not the extraneous “black” portion of the image, go to the tools on the left hand side and select “elliptical marquee tool” under marquee tool and click and drag on the image to draw a dotted line circle as big as the required area (Figure 6); the area within the dotted circle is now selected. Once selected, right click on it to get further options; then click on “inverse selection” to select the extraneous portion of the image and hit “delete” key on the keyboard to obtain only the required area of the photomicrograph which is now ready to be merged (Figure 7). “Save” this file in psd (Photoshop) or “TIFF” format by clicking on “file” and selecting “save as.” Other formats are not opted to avoid image compression, which can seriously alter the resolution.

Repeat the above sequence of commands to all of the photomicrographs of the entire section and crop. “Batch cropping” is also possible in Adobe Bridge itself, thereby reducing much time, but the disadvantage of Adobe Bridge here is the fact that cropping can be done only in rectangular shapes and not in circles. This may lead to missing out some corners of section in the final whole slide image. If one is using a microscope attached with a dedicated camera (e.g., Jenoptik camera), all the photomicrographs can be directly optimized and merged. The additional step of nondestructive cropping is not required as there is no such “black extraneous portion” in the photomicrographs.

#### 2.2.2. Merging the Optimized Image

All optimized images will be merged to obtain a collage of photomicrographs.

**Figure 8:**
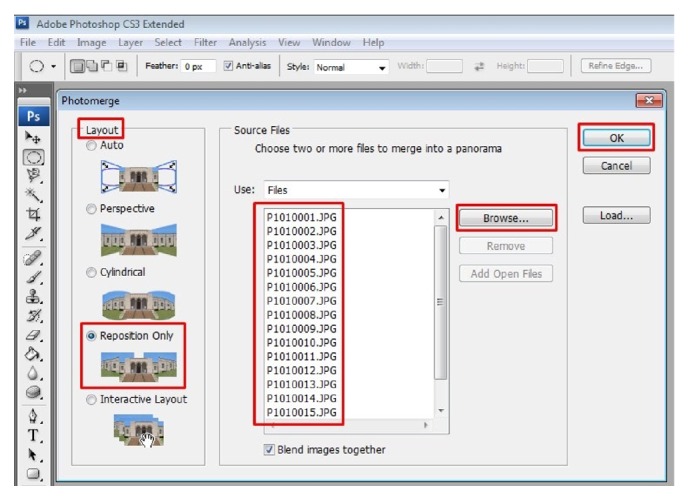
Go to file > automate > photo merge to actually merge. Browse files from folder where the optimized files are stored and select all and hit OK. Note the red boxes in the figure. Follow them for an easy workflow.

**Figure 9:**
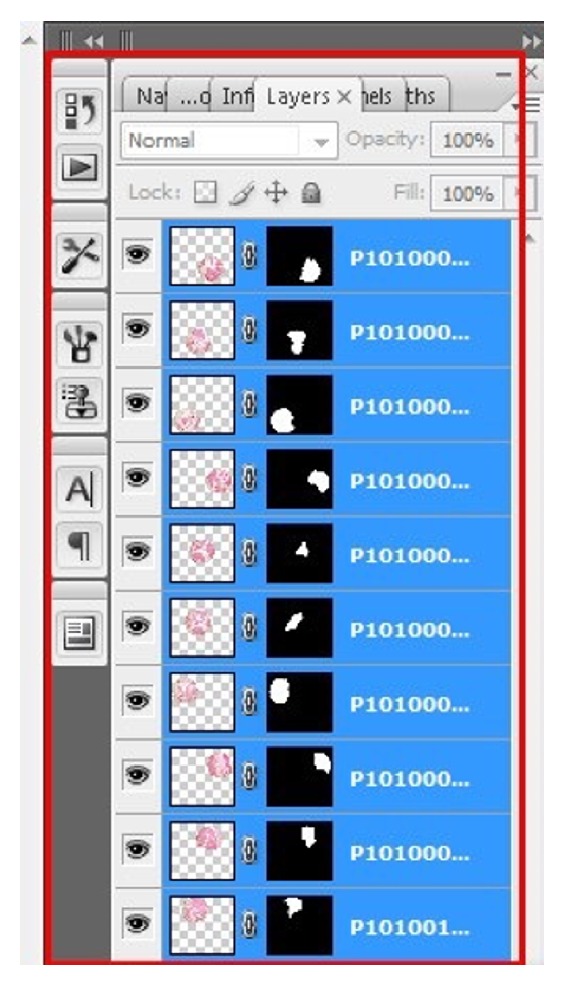
On “photomerge” command, notice how the software integrates multiple layers and stitches into one. One can observe this on layers panel. Note the red boxes in the figure. Follow them for an easy workflow.

**Figure 10:**
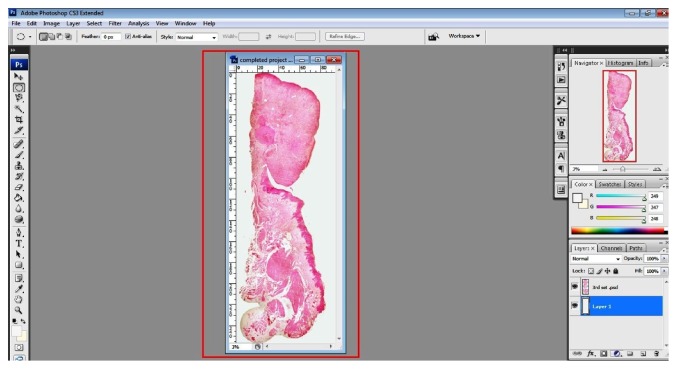
The final “stitched” image of multiple microphotographs. Minor adjustments like background color are applied to make it more uniform and appealing. Note the red boxes in the figure. Follow them for an easy workflow.

Once the images are optimized and saved, they have to be merged to obtain a seamless collage of microphotographs which are perfectly aligned and merged to create a high resolution digital image of the entire section. To do this, go to “file” and select “automate” to get further options in drop down menu. Select “photomerge” option to obtain a pop-up window on photomerge (Figure 8). Select “reposition only” layout by clicking on it. For source files, browse the folder where all optimized images are stored and select all to get all images in the list (Figure 8). Hit “Ok” to obtain a beautiful panoramic image of an entire section. During the process of actual merging one can actually see how software aligns and blends itself numerous “circles” of microphotographs and creates a panorama (Figure 9). After blending all the images using photomerge command one can see the high resolution image of entire tissue section (WSI) (Figure 10). The obtained image consists of many layers which can be flattened to a single layer by going to “layer” tab and select “merge down” under drop down menu. Once merged the image can be stored in “TIFF” or Photoshop format to maintain resolution.

*User Interface*. Once flattened, created digital image of whole slide can be viewed on computer interface using Zoomify (discussed later).

## 3. Discussion

A virtual slide (VS) is composed of a collection of digital images representing a histological/cytological slide at all magnification levels together with all relevant clinical data. These can then be viewed on a computer by means of an interface (“user-friendly”) that allows one to select the more appropriate fields and to examine them at different magnifications, rapidly going from panoramic views to high resolution and vice versa. In comparison with glass slides, VSs have several advantages arising from their digital nature and can be considered a common platform for a wide range of applications [11]; also its ability to examine images at different magnifications as well as to view histology and immunohistochemistry side by side on the screen [5] makes it interesting. It would also permit annotation of the virtual slides [5].

The VM system is a realistic alternative, in terms of its ability to mimic a conventional microscopy [4] in most aspects. In our study, we tried building an economical alternative to whole side scanner by using a common digital camera mounted on a regular trinocular microscope in conjunction with most coveted image editing software Adobe Photoshop.

Usually digitizing an entire slide using a fully automated whole slide scanners takes up to 8 hours. If the image of each field is compressed at an appropriate quality level (a compression ratio of, say, 35 : 1) it requires about 40 Kbytes to be stored, resulting in a total storage requirement of about 600 megabytes per slide. Thus one CD-ROM can be used to store one virtual slide [6]. We, in our system of using Adobe Photoshop, worked approximately 2 hours to digitize one slide (it depends on the hardware specifications of computer used). Each digital slide created using Adobe Photoshop by us also occupies about 500–600 megabytes of hard disc space corresponding to one CD-ROM. So we can hypothesize that our system is similar to dedicated scanners in terms of storage parameter. But expensive ultrarapid scanners like the “Dmitri virtual slide processor” have been found to reduce the virtual slide processing cycle more than 10-fold, as compared with other virtual slide systems [12]. Recently introduced advanced models of scanners are capable of scanning the slide within minutes but are hugely expensive.

Our Photoshop method of creating virtual slide needs no much technical knowledge. In today's digital world, most of histopathologists have a basic knowledge on a “digital image” and their different formats. Though one might have to try out many times before being actually able to master this technique. In strict sense with the “photomerge” command one can expect a perfect virtual slide, but, in reality, a less than perfect image is usually created. This depends upon many factors like source of image and its clarity and appropriate cropping of the extraneous part of the image; microphotographing all the areas without missing even small portion is the most important. We propose a near perfect virtual slide that can be definitely created provided that all guidelines are followed with interest, dedicated effort, time, and patience.

**Figure 11:**
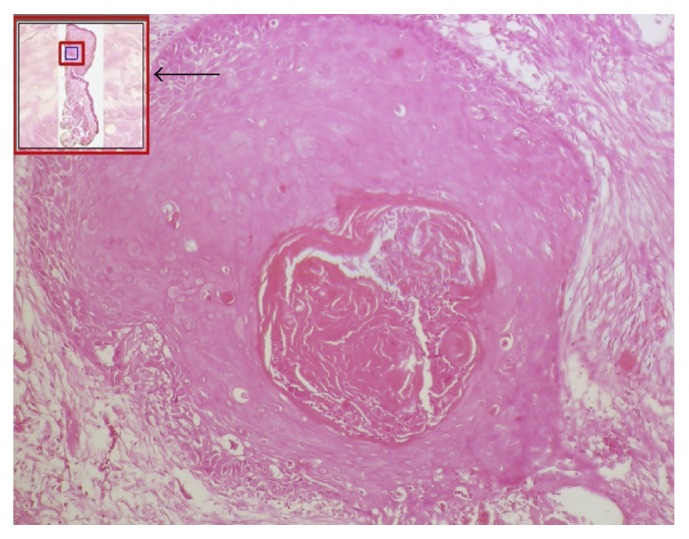
User interface in Zoomify. One can zoom in and out at high resolution. Note an enlarged view of small boxed area within a navigator (large red box with arrow). Note the red boxes in the figure. Follow them for an easy workflow.

*Zoomify User Interface*. The so-created virtual pathology slide using Adobe Photoshop can be observed in high resolution by zooming-in in Photoshop itself, or best they can be exported as an external TIFF file or exported it as a “Zoomify.” This file can be opened using a web browser that has an interface to be able to zoom in and move around different parts of the image, just like scanning a slide under a microscope. To do this, go to file > export > Zoomify. Zoomify is an external product and what Photoshop offers is just a simplified version. The created image can thus be shared with anyone on internet or intranet. Ways to share large images on internet are variable; they are beyond the scope of this article to describe. When exported as Zoomify, one can have multiple options like navigator window, background color, size of the presentation window and quality of image, and so forth (Figure 11). It takes some trial and error to obtain the best possible settings, and it is fun to play with also.

Future potentials of WSI are immense. WSI can be simply used during comparison with multiple stains side by side; if multiple sections stained using multiple stains have to be compared, one can use “match and zoom” tool in Adobe Photoshop to compare the exact areas in the section. If any researcher wants to identify and count specific type of cell(s), this can be done using the entire section rather than the conventional method of counting the cells in “per high power fields.” Several recent studies of The Cancer Genome Atlas data have illustrated important relationships between morphology observed in whole slide images, outcome, and genetic events [13]. Numerous studies using WSI relating to Identification of mitosis [14–16] are already published. Ultimately, we believe that it comes down to individual's intelligence and creativity to show how one can make use of such high resolution histopathological images. This is not without challenges; one may face problems in workflow integration, technological infrastructure, pathologist acclimatization, global standardization for clinical practice, and cost factors among others [17].

Romer et al. in 2003 worked on using a modified standard microscope to generate virtual slide by adding a scientific robotic stage with a controller and a camera for precise capturing of all the microscopic fields. This technique produced high quality images from tissue sections including tissue arrays [18].

This technique of ours is possible at almost “negligible” cost. But it comes with certain limitations like absolute requirement of precise microphotographs, patience, repeated trial and errors, high end specification computer with a lot of hard disc space and high memory and a highly interested, dedicated personnel. The created whole slide image will be of huge size each slide running in hundreds of megabytes to gigabytes if high resolution is required. So storage would be another problem if it is routinely done for archiving purpose.

## 4. Conclusion

Considering the “cost” factor of dedicated slide scanners it looks that none of the developing countries can afford and incorporate them into their routine diagnostics and pathology training. So we propose that this technique of producing enlarged field of view of stitched high resolution images using Adobe Photoshop as an economical alternative to obtain high resolution WSI.

We do agree that it is slightly time consuming when compared to ultrarapid dedicated scanners, but when we weigh the time-cost ratio, this can be seriously helpful to many pathologists and limited need of such whole section image can make this technique an economical alternative. Also, the storage of large size digital images might be a problem for archiving purpose. However, these problems are likely to be overcome by technological advances in the future.

## Supplementary Material

A step by step video depicting the process of merging numerous photomicrographs captured using basic camera-trinocular microscope set up using Adobe Photoshop.
